# L-arginine metabolism in breast cancer: mechanisms and therapeutic targets

**DOI:** 10.3389/fonc.2026.1844871

**Published:** 2026-06-12

**Authors:** Antonia Röglin, Rainer Böger, Juliane Hannemann

**Affiliations:** Institute of Clinical Pharmacology and Toxicology, University Medical Center Hamburg-Eppendorf, Hamburg, Germany

**Keywords:** breast cancer, breast cancer subtypes, enzyme inhibition, L-arginine metabolism, L-arginine starvation, L-arginine supplementation, therapeutic targets

## Abstract

Breast cancer is the most frequently diagnosed cancer and the leading cause of cancer-related death among women worldwide. It is a highly heterogeneous disease that can be divided into different molecular subtypes, which differ in morphology, prognosis, and treatment response. While metabolic reprogramming is known to support tumor growth and survival, the specific role of L-arginine metabolism in breast cancer is not completely understood. L-Arginine is a semi-essential amino acid, it is involved in key pathways regulating cell proliferation, immune response, and protein metabolism. Dysregulation of L-arginine-related enzymes, such as arginases and nitric oxide synthases, can influence tumor growth, metabolic signaling, and the interaction between cancer and immune cells. Therapeutic targeting of L-arginine metabolism has been explored by three main approaches: L-arginine starvation, L-arginine supplementation, and enzyme inhibition. However, results have remained inconsistent, possibly due to differences in metabolic adaptations across molecular breast cancer subtypes. This review provides an overview of current knowledge on L-arginine metabolism in breast cancer, including metabolic reprogramming, subtype-specific regulation, and therapeutic opportunities. It also discusses challenges and highlights the need for further research to define subtype-specific regulation of L-arginine metabolism.

## Introduction

Breast cancer (BC) is the most prevalent cancer in women, with a global incidence of 2.3 million cases in 2022 (1). The mortality rate in 2022 exceeded 670,000 (1). Breast cancer is recognized as a group of diseases instead of a single disease, and it can be divided into different subtypes, which differ in morphology, biology and clinical outcome (2). In clinical practice, breast cancer is classified based on the immunohistochemical expression of estrogen receptor (ER), progesterone receptor (PR), and human epidermal growth factor receptor (HER2). These clinical subtypes are associated by differences in relapse-free and overall survival and guide treatment decisions (3, 4). At the molecular level, microarray techniques are used to distinguish between four commonly accepted intrinsic subtypes of breast cancer (**Table 1**): luminal A, luminal B, HER2-enriched, and triple-negative breast cancer (TNBC) (3, 13).

**Table 1:** Characteristics of breast cancer subtypes.

Subtype	Luminal A	Luminal B	HER2-enriched	Triple-negative	Reference
Receptor status	ER+PR+HER2-	ER+PR+HER2+/HER2-	ER-PR-HER2+	ER-PR-HER2-	(5–7)
Frequency	50%	15%	20%	15%	(7–9)
5-year survival rate	95.6%	91.8%	86.5%	78.4%	(10)
Therapy	Endocrine therapy	Endocrine therapy/Chemotherapy	Endocrine therapy/Chemotherapy/HER2-targeted therapy	Chemotherapy	(2, 11, 12)

ER, estrogen receptor; PR, progesterone receptor; HER2, human epidermal growth factor receptor.

Luminal A tumors (ER+/PR+, HER2−) exhibit low clinical grade, have a good prognosis and are primarily treated with endocrine therapy (2, 5, 10–12). Luminal B tumors (ER+/PR+, HER2±) are more aggressive, have worse outcomes and can be treated by endocrine therapy, chemotherapy, and HER2-targeted agents (2, 5, 14–16). HER2-enriched cancers (ER−/PR−, HER2+) are highly aggressive due to breakdown of cell-cell junctions promoting migration, but HER2-targeted therapies (trastuzumab) have significantly improved survival (2, 6, 7, 16). The TNBC subtype (ER−/PR−/HER2−) is characterized by rapid progression, early metastasis, and poor prognosis. Due to the lack of targeted treatment options, TNBC is the most challenging BC subtype to treat and chemotherapy often remains the only treatment option (2, 3, 7, 17–19).

Furthermore, therapeutic resistance represents a major challenge in breast cancer management. Resistance can be classified as intrinsic, where tumors are resistant from the beginning of therapy, or acquired, in which resistance develops during treatment. Resistance can reduce therapeutic efficacy and may result in relapse or metastasis in some patients (20, 21). Consequently, the development of novel and personalized treatment options is important to improve outcomes for breast cancer patients.

A hallmark of cancer is metabolic reprogramming, which includes, pathways of energy metabolism, which are reprogrammed from oxidative phosphorylation to glycolysis (“Warburg effect”) (22). Metabolic reprogramming may lead to dependencies and vulnerabilities in tumor cells, which can be exploited in therapy. One approach to find new therapeutic options is targeting L-arginine metabolism, as cancer cells rely on L-arginine for protein synthesis, nitric oxide signaling, and cell proliferation (23). This review summarizes current knowledge on L-arginine metabolism and its role in breast cancer. Furthermore, various therapeutic opportunities targeting the L-arginine metabolism, including L-arginine starvation and L-arginine supplementation, as well as enzyme inhibitors are discussed.

## L-arginine metabolism

**Figure 1:**
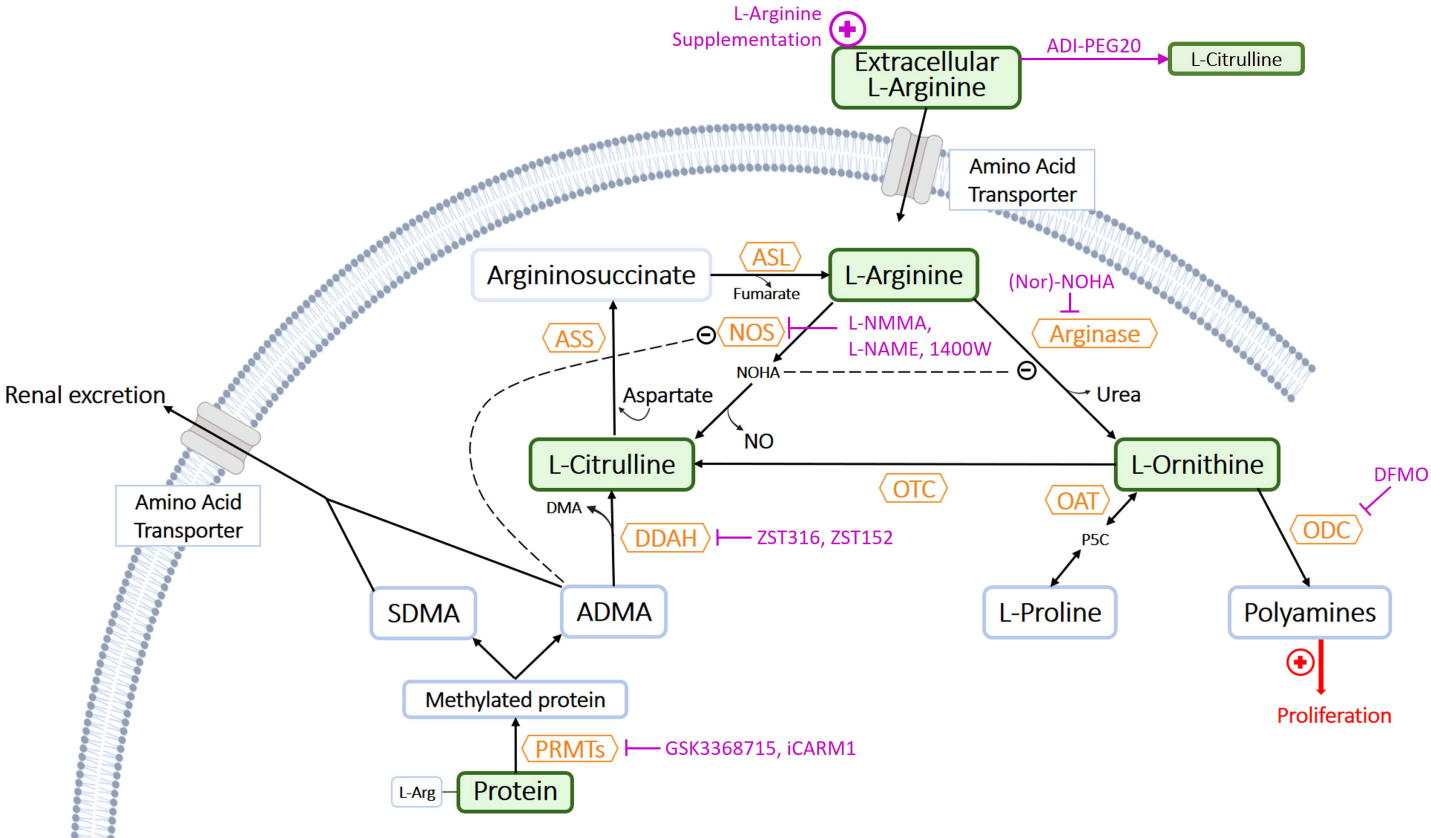
Schematic overview of L-arginine metabolic pathways. L-Arginine is transported into cells via amino acid transporters or synthesized from L-citrulline. L-Arginine can be converted into L-citrulline and NO or into L-ornithine and urea. L-Ornithine then acts as a precursor for polyamines which are important for cell proliferation. Potential therapeutic targets and their associated drugs are highlighted in purple. This illustration was created with BioRender.com. 1400W, N′′[[3-(aminomethyl)phenyl]methyl]-ethanimidamide; ADI-PEG20, pegylated arginine deiminase; ADMA, asymmetric dimethylarginine; ASL, argininosuccinate lyase; ASS, argininosuccinate synthase; DDAH, dimethylarginine dimethylaminohydrolase; DFMO, difluoromethyl-ornithine; DMA, dimethylarginine; iCARM1, inhibitor of coactivator-associated arginine methyltransferase 1 (protein-L-arginine-methyltransferase 4 inhibitor); L-NAME, N^5^-[imino(nitroamino)methyl]-L-ornithine methyl ester; L-NMMA, NG-monomethyl-L-arginine; NO, nitric oxide; NOHA, Nω-hydroxy-L-arginine; nor-NOHA, Nω-hydroxy-nor-L-arginine; NOS, nitric oxide synthase; OAT, ornithine aminotransferase; ODC, ornithine decarboxylase; OTC, ornithine transcarbamylase; P5C, pyrroline-5-carboxylate; PRMT, protein-L-arginine-methyltransferase; SDMA, symmetric dimethylarginine.

L-Arginine (2-amino-5-guanidino-pentanoic acid) is a dibasic, proteinogenic amino acid which can be obtained through dietary intake or from endogenous synthesis from L-citrulline and through protein turnover in various cell types (24, 25). In healthy adults, endogenous synthesis is generally sufficient to meet physiological requirements. Under conditions of catabolic stress, including inflammation, infection, or in cases of kidney or small intestine failure, endogenous synthesis may be insufficient to sustain metabolic demands. L-Arginine was first classified as semi-essential amino acid in 1937 based on the experimental finding that endogenous synthesis of L-arginine was not sufficient for normal growth in young rats (26, 27). L-Arginine is involved in protein metabolism and in the synthesis of creatine, L-glutamate, L-ornithine, nitric oxide (NO), and polyamines (23). In these pathways, L-arginine acts as a substrate for nitric oxide synthases (NOS) and arginases (**Figure 1**).

### L-arginine – L-citrulline pathway

L-Arginine is metabolized by NOS to L-citrulline and NO, via the intermediate N^ω^-hydroxy-L-arginine (NOHA). Three isoforms of NOS have been identified, with names derived from the cell type they were initially isolated from: neuronal NOS (nNOS, NOS I), inducible NOS (iNOS, NOS II) and endothelial NOS (eNOS, NOS III) (28, 29). nNOS plays a critical role in regulating memory formation and neuronal signaling involved in pain perception, yet it is also expressed in other tissues like the epithelia (29). The second isoform, iNOS, can be found in macrophages and other immune cells. The naming is based on the fact that the mRNA of this isoform is upregulated upon cytokine stimulation. When induced, iNOS produces NO at high rates (28). eNOS is expressed in endothelial cells and is essential for the health of the vascular system, as it contributes to the maintenance of physiological blood flow (30). iNOS and eNOS expression have been detected in breast cancer tissue (31–33) and in several tumor cell lines (34, 35).

### L-arginine – L-ornithine pathway

The enzyme arginase catalyzes the metabolism of L-arginine to L-ornithine and urea. This pathway is crucial for the urea cycle and is key to biochemical pathways that are essential for cell proliferation (36, 37). Two isoforms of arginase are known that differ in their organ and subcellular location: Arginase-1 is a cytosolic enzyme mainly localized in the liver, while the second isoform, arginase-2, is an extrahepatic form localized within the mitochondrial matrix (30). Given their different location, both isoforms have distinct tasks. Arginase-1 is primarily involved in urea synthesis and ammonia detoxification. Arginase-2 synthesizes L-ornithine and urea and, given its location in the mitochondria, influences metabolic adaptation and immune regulation (38). L-Ornithine is further converted into polyamines by ornithine decarboxylase (ODC) or into L-proline by ornithine aminotransferase (OAT). By binding DNA and altering the structural makeup of chromatin, polyamines are known to modulate gene expression and thereby promote cell development (39).

### L-arginine recycling pathway

Numerous different cell types can endogenously synthesize L-arginine from L-citrulline. Argininosuccinate synthetase (ASS) and argininosuccinate lyase (ASL) are responsible for the conversion of L-citrulline to L-arginine. Their expression can be induced by inflammatory cytokines, lipopolysaccharides, and endoplasmic reticulum stress (40–42). Oftentimes, this induction happens in parallel to the up-regulation of iNOS, allowing some of the L-citrulline produced by NOS to be recycled into L-arginine. The recycling of L-citrulline to L-arginine requires the sequential interaction of the enzymes ASS and ASL. ASS catalyzes the ATP-dependent formation of argininosuccinate from L-citrulline and L-aspartate, which is then metabolized by ASL to generate L-arginine and fumarate (30). Once induced, the recycling pathway has been found to be crucial for maintaining adequate intracellular L-arginine concentrations to meet increased metabolic requirements in breast cancer progression (41) and in immune responses (43).

### Transmembrane transport of L-arginine

In addition to endogenous synthesis and dietary sources, the intracellular concentration of L-arginine is influenced by the capacity and efficiency of L-arginine transporters expressed in the plasma membrane (44). Because amino acids are hydrophilic, they cannot freely transfer through the mammalian cell membrane and thus rely on specific transport systems (45). L-Arginine uptake from the extracellular space is mediated by members of the solute carrier 7 family (SLC7), particularly the cationic amino acid transporters (CATs) [reviewed in (46)]. The classification of transporters is based on amino acid substrate specificity, sequence similarity, and ion dependence (47). While ion-dependent transporters use ion motive force to accumulate amino acids in the cell, ion-independent transporters transfer amino acids according to their electrochemical gradient (48). The Na^+^-independent cationic amino acid transporter (CAT) family, which includes CAT-1, CAT-2, CAT-3, and CAT-4, is the most common family of L-arginine transporters (49–51). CAT-1 and CAT-2 are further known to transport the L-arginine derivatives asymmetric dimethylarginine (ADMA) and symmetric dimethylarginine (SDMA) (52, 53). In addition to the CAT transporters, heterodimeric amino acid transporters, such as transporters encoded by SLC7A6 and SLC7A7, belong to the 7^th^ group of solute transporters. SLC7A6 and SLC7A7 each encode for a membrane protein that acts as the light subunit in the formation of a heterodimer with the glycoprotein 4F2 as heavy subunit (encoded by SLC3A2). These are then referred to as the y^+^L transport system, which transports dibasic amino acids out of epithelial cells in exchange for neutral amino acids and Na^+^ (54). In the absence of Na^+^, intracellular dibasic amino acids are exchanged for extracellular dibasic amino acids (55). For L-arginine, this may predominantly result in efflux under conditions with high extracellular neutral amino acids (e.g., glutamine) (56, 57). Furthermore, L-arginine can be transported by the ATB^0,+^ transporter (encoded by SLC6A14), which mediates the cellular uptake of L-arginine, L-glutamine, and all essential amino acids in a sodium- and chloride-dependent manner (58).

### Regulation of methylated arginines

Protein-L-arginine-methyltransferases (PRMTs) catalyze the post-translational methylation of arginine residues in proteins, first generating N-monomethylarginine (MMA) and subsequently converting MMA to the dimethylated amino acid derivatives: Asymmetric (ADMA) and symmetric dimethylarginine (SDMA) (29, 59, 60). PRMTs are divided into type I enzymes, producing ADMA and MMA; type II enzymes, producing SDMA and MMA; and type III PRMTs, producing only MMA [reviewed in (61)]. L-Arginine methylation by PRMTs is a key post-translational modification affecting gene expression, protein function, cell signaling, and proliferation (62–65). During protein turnover, methylated arginine residues are released into the cytosol. SDMA can be cleared by renal excretion and metabolized by alanine-glyoxylate aminotransferase 2 (AGXT2) (66, 67), whereas ADMA is primarily degraded intracellularly by dimethylarginine dimethylaminohydrolase (DDAH), metabolized by AGXT2, or cleared through renal excretion (68). ADMA is known as an endogenous inhibitor of NOS, and elevated levels of ADMA reduce NO production (69–71). SDMA does not directly inhibit NOS, but like ADMA, it may interfere with L-arginine transport, since it competes with L-arginine for transport via CAT-1 and CAT-2, and may thus indirectly influence NO synthesis (72).

DDAH hydrolyses ADMA to L-citrulline and dimethylamine, thereby regulating ADMA levels and contributing to the maintenance of NO production (73). There are two isoforms, DDAH1 and DDAH2, which have distinct tissue expressions. DDAH1 is mainly found in liver and kidney, while DDAH2 is membrane-associated and predominant in endothelial cells (74). Due to their interaction with the NO pathway, ADMA and SDMA are involved in a variety of human diseases, including chronic renal failure, cardiovascular diseases, diabetes, pulmonary hypertension, and chronic lung diseases (69, 75–78).

### Relevance of L-arginine metabolism for cell proliferation

L-Arginine metabolism has significant relevance for cell proliferation through several mechanisms. It serves as a critical nutrient for protein synthesis and energy metabolism in rapidly dividing cells, including immune and tumor cells. By providing substrate for protein synthesis and the production of polyamines, it promotes cell proliferation and tissue growth (79). L-Arginine is also essential for T cell proliferation, differentiation, and survival. Studies have shown that elevated intracellular L-arginine induces metabolic changes in activated T cells, including a shift from glycolysis to oxidative phosphorylation. This shift improves metabolic fitness and survival of T cells (80–82). When L-arginine is limited, cells may undergo growth arrest and fail to progress through the cell cycle. Previous studies have reported that L-arginine deprivation inhibits proliferation and induces apoptosis in immune cells, including T cells (43). In the breast cancer cell line MCF-7, reduced metabolic activity and protein synthesis have been observed after L-arginine depletion (83). Together, these findings indicate that L-arginine metabolism is a relevant regulator of cell proliferation, although its effect on cell proliferation may vary in different contexts and across different cell types.

## Role of L-arginine metabolism in breast cancer

It is widely recognized that metabolic changes play a crucial role in cancer development and in the progression of tumors. Therefore, gaining a comprehensive understanding of metabolic alterations occurring during cancer development in breast tissue might aid in identifying potential biomarkers and drug targets linked to cancer development and progression. Previous studies have linked metabolic vulnerabilities of breast cancer to L-arginine metabolism (41, 84–86), suggesting it as a promising area for further research.

### Metabolic reprogramming

Cancer progression is often accompanied by a disturbance of L-arginine bioavailability (87, 88). Some cancer cells can be considered L-arginine auxotrophic, as they lack expression of ASS1 (89). These cells are unable to synthesize L-arginine internally and rely on external L-arginine supply. In TNBC, ASS1 knockdown and knockout promoted cell proliferation *in vitro* and *in vivo*, suggesting ASS1 acts as a tumor suppressor in TNBC (90). In line with this, some studies have reported that low or absent ASS1 protein expression is associated with poor survival and increased recurrence in breast cancer cohorts (84, 91). However, other analyses, which evaluated datasets of the Cancer Genome Atlas (TCGA) and Gene Expression Omnibus (GEO) reported that high ASS1 gene expression is associated with poor overall survival in breast cancer, even though ASS1 gene expression is generally lower in cancerous tissue compared to normal tissue (92, 93). These varying results might be due to the fact that gene expression does not necessarily correlate directly to protein expression. Low translational efficiency, post-transcriptional regulation, or altered enzymatic activity may lead to low ASS1 protein levels or low ASS1 functionality, even if gene expression of ASS1 is high. Future studies examining ASS1 gene and protein expression, as well as enzymatic activity, in relation to prognosis in breast cancer patients might help to clarify the association between ASS1 and prognosis.

According to recent experimental and clinical studies, arginase is highly expressed in breast cancer (94–96). Elevated arginase activity was detected in the serum of breast cancer patients, which also correlates with cancer progression stages (97). After chemotherapy, arginase activity was found to decrease, implying its potential as a biomarker for disease progression and treatment response (97). Furthermore, increased polyamine synthesis has been found in breast cancer (98). Reduced spermine content, decreased cell proliferation, and induced apoptosis have been shown as a result of pharmacological arginase inhibition in breast cancer cells with elevated arginase activity (39). Additionally, studies have shown that arginase-2 is the predominant isoform expressed in breast cancer tissues and cell lines (39, 94, 95). While 80% of breast tumor samples analyzed by Singh et al. (39) showed arginase-2 expression, arginase-1 was expressed in only 50% of the breast tumor samples at lower levels.

**Figure 2:**
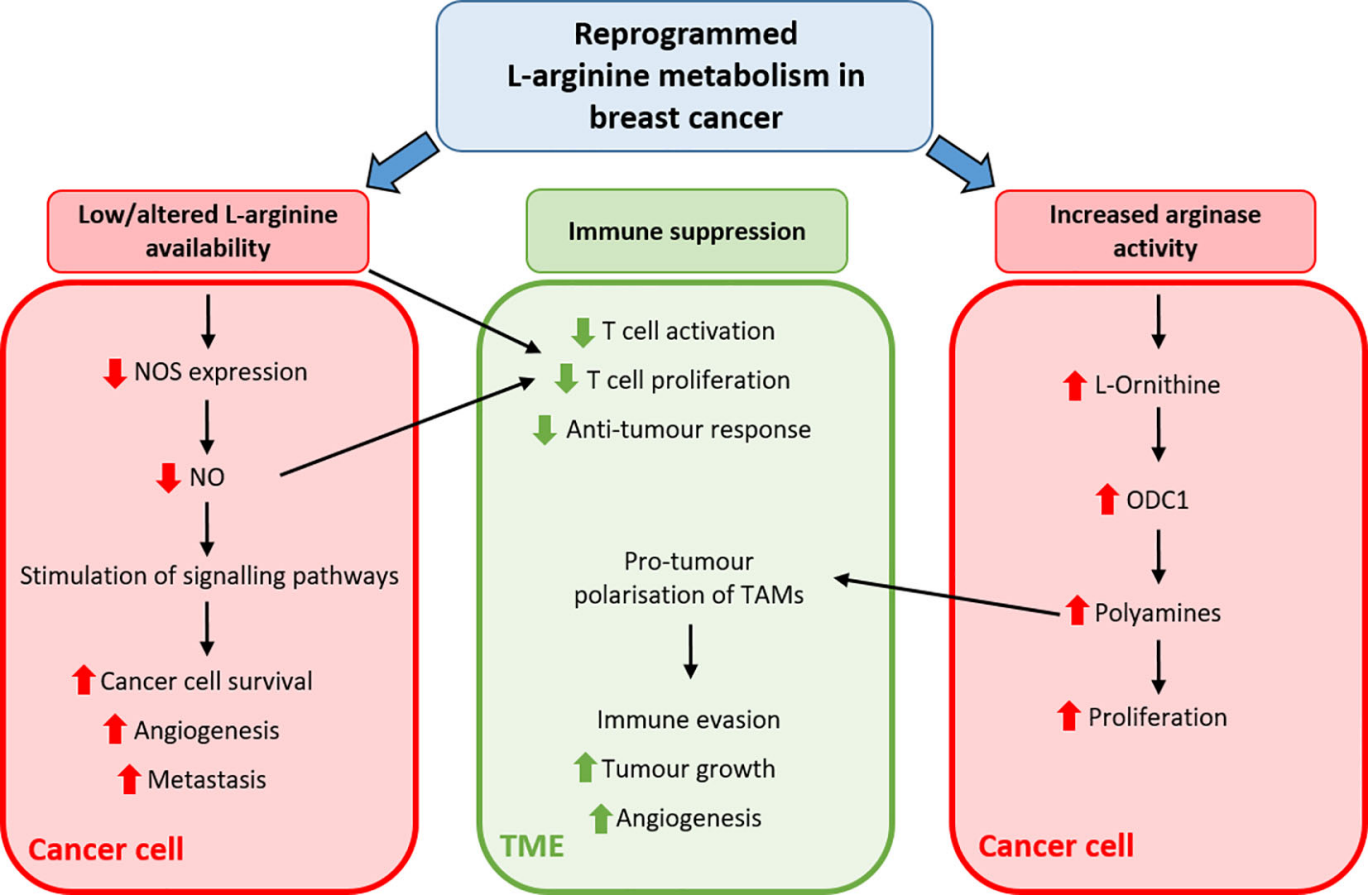
Schematic overview of selected mechanisms involved in reprogrammed L-arginine metabolism in breast cancer. L-arginine plays a dual role in breast cancer, supporting both tumor-promoting metabolic pathways and anti-tumor immune functions. Low or altered L-arginine availability and increased arginase activity promote tumor progression through multiple mechanisms. Reduced NO production enhances cancer cell survival, angiogenesis, and metastasis, while increased arginase activity drives polyamine synthesis and proliferation. In the tumor microenvironment, these alterations suppress T cell activation and proliferation, leading to immune evasion and pro-tumor macrophage polarization. NO, nitric oxide; NOS, nitric oxide synthase; ODC, ornithine decarboxylase; TAM, tumor-associated macrophage; TME, tumor microenvironment.

NOS competes with arginase for L-arginine as a substrate (99). Excessive arginase activity in myeloid-derived suppressor cells depletes L-arginine in the tumor microenvironment, which reduces substrate availability for iNOS-mediated NO production and thereby impairs T cell responses (**Figure 2**) (100). This promotes further growth of tumor cells. Numerous physiological processes are regulated by NO, which may have pro- and anti-tumor effects, depending on its concentration (101–104). When NO concentrations are low, different important signaling pathways for tumor cell survival can be stimulated, including protein kinase B (AKT) and mammalian target of rapamycin (mTOR) (105). This may lead to the stimulation of cancer progression, avoidance of apoptosis, and increased angiogenesis and metastasis (101). By contrast, high levels of NO may cause cellular cytotoxicity to cancer cells by causing DNA damage, oxidative stress, and apoptosis (106). A recent study reported significantly higher arginase activity and lower NO levels in the saliva of breast cancer patients compared to the control group (107). The study also showed that an increased arginase:NO ratio is characteristic of the early stages of breast cancer, highlighting its potential as a diagnostic marker (107). Variation of arginase and NO in the saliva of breast cancer patients suggests broader, systemic alterations of the L-arginine metabolism. The regulation of L-arginine metabolism is known to be influenced by cytokines (108). Cytokines are signaling molecules that are produced by immune cells and are important for immune responses and inflammation (109, 110). In the serum of breast cancer patients, cytokine concentrations are altered, with most of the cytokines having significantly higher concentrations in breast cancer patients than healthy individuals (111–113). Arginase and NOS activities can be induced by T helper cell type 1- and T helper cell type 2-type-derived cytokines, which are responsible for controlling cellular and humoral immune responses, respectively (108). Therefore, L-arginine metabolism is likely regulated by systemically acting molecules, such as cytokines, which can lead to alterations in peripheral fluids such as saliva. Furthermore, as arginases and NOS compete for L-arginine, they regulate the metabolic flux between polyamine synthesis and nitric oxide production. This balance represents an important metabolic control node that may influence tumor growth and immune responses.

### Metabolic switch and L-arginine signaling

A metabolic switch is a hallmark of cancer progression. It helps cancer cells to adapt to nutrient-poor environments and supports rapid tumor growth (114). Metastasis formation usually occurs in advanced stages of cancer. However, early detection of metastases through plasma biomarkers may guide treatment and improve the prognosis of patients. In early phases of metastasis, an increase in arginase activity and polyamine synthesis, associated with arginase-dependent immunosuppression and polyamine-dependent cell proliferation was observed in a breast cancer mouse model (22). During advanced metastatic phases, breast tumors reprogram the energy metabolism from mitochondria-mediated oxidative phosphorylation towards glycolysis (“Warburg effect”) and the pentose phosphate pathway (22). In breast cancer progression, glycolysis is induced by the phosphatidylinositol 3-kinase (PI3K)/protein kinase B (AKT)/mammalian target of rapamycin (mTOR) signaling pathway (115), which is of great importance for cell proliferation, cell metabolism, apoptosis, and angiogenesis (116). Approximately 26% of breast tumors have a mutation of the PIK3CA gene, with higher frequencies (>30%) observed in hormone-receptor-positive, HER2-negative patients (117). This mutation induces the hyperactivation of PI3K and thereby promotes cancer progression (116). L-Arginine can directly activate mTOR, a nutrient-sensing kinase, through three different ways, which have been described in detail elsewhere (118). The activation of mTOR then promotes anabolic processes, such as protein synthesis, *de novo* lipid synthesis, and nucleotide synthesis, which lead to increased cellular proliferation and tumor growth (118). The exact mechanisms by which mTOR activation leads to such anabolic processes are highly complex, and mechanistic details have been thoroughly described in another comprehensive review (119). Therefore, L-arginine metabolism and PI3K/AKT/mTOR signaling are tightly linked, with L-arginine availability influencing the mTOR pathway and thus affecting breast cancer progression.

### Tumor microenvironment

L-Arginine metabolism plays a multifaceted role in the breast cancer tumor microenvironment (TME), impacting both cancer cells and immune cells (**Figure 2**) (118, 120, 121). Metabolic reprogramming of L-arginine metabolism in cancer often leads to altered L-arginine availability within the TME. Breast cancer cells and immune cells compete for L-arginine, and increased arginase activity of breast cancer cells depletes L-arginine locally. This depletion limits L-arginine availability for T cells and impairs T cell activation, T cell proliferation, and anti-tumor responses (122). At the same time, increased arginase activity in tumor cells promote L-ornithine and polyamine synthesis and thus facilitates tumor growth (123, 124). Arginase-2 is the predominant isoform expressed by breast cancer cells, whereas arginase-1 is highly expressed in myeloid-derived suppressor cells and tumor-associated macrophages (TAMs), where it mediates local L-arginine depletion and immunosuppression (125).

A recent study showed that the metabolic interplay between cancer cells and TAMs is crucial for L-arginine-driven breast cancer progression (126, 127). L-Arginine metabolism promotes a pro-tumor polarization of TAMs, which suppresses CD8^+^ T cell cytotoxicity. In this process, spermine was identified as a central mediator by promoting pro-tumor TAM polarization through epigenetic regulation involving p53/TDG-dependent DNA demethylation. Hence, polyamines derived from L-arginine metabolism reinforce polarization of TAMs, which in turn facilitates immune evasion and tumor growth (126). In summary, L-arginine metabolism supports tumor cell proliferation and immune evasion in the breast cancer TME, and its dysregulation drives both metabolic and immunological changes to promote cancer progression.

### Subtype-specific differences in regulation of L-arginine metabolism

Emerging evidence demonstrates that L-arginine metabolism is regulated differently in the various breast cancer subtypes. Different subtypes of breast cancer exhibit distinct expression patterns of key enzymes involved in the metabolism of L-arginine. This leads to varying concentrations of L-arginine and its metabolites within tumor cells and in patient plasma. For example, a translational study by our group has shown that hormone receptor-positive cell lines (MCF-7 and BT-474) had significantly higher L-arginine concentrations, and arginase-2 expression was highest in BT-474, MDA-MB-468 and MDA-MB-231 cells. BT-474 and MDA-MB-468 cells also exhibited high concentrations of L-ornithine, and the TNBC cell line MDA-MB-468 showed an elevated L-ornithine/L-arginine ratio, indicating increased arginase activity. Furthermore, ADMA concentrations were highest in patients with TNBC and lowest in HER2-enriched patients, which was similar in the breast cancer cell lines. Moreover, in patients with luminal A and triple-negative breast cancer, ADMA and L-citrulline were associated with recurrence and total mortality, respectively (128).

In addition, there is evidence that ASS1 expression is heterogeneous across breast cancer subtypes and cell lines. MCF-7 cells were shown to have relatively high ASS1 expression, while MDA-MB-231 cells show lower levels (91). In MDA-MB-231 cells, it was also demonstrated that ASS1 knockdown increased cell proliferation, whereas ASS1 overexpression led to an inhibition of cell proliferation (91). This is consistent with the results of the study by Lou et al. (90), in which ASS1 knockdown led to increased cell proliferation in MDA-MB-468 cells, a finding that was also observed in an MDA-MB-468 xenograft ASS1 knockout model (90). These findings suggest that ASS1 may act as a tumor suppressor.

In TNBC, an overexpression of iNOS has been observed, with significantly elevated mRNA levels in comparison to non-TNBC subtypes, and this correlated with reduced overall survival (32, 129, 130). NO derived from iNOS promotes tumor progression via different mechanism including activation of PI3K/AKT, epidermal growth factor receptor, and mitogen-activated protein kinase, as comprehensively reviewed by Basudhar et al. (131). In contrast, HER2-enriched breast cancer, appeared to have alterations in the polyamine synthesis pathway. HER-enriched tumors showed elevated levels of acetylated polyamines and elevated expression of spermidine/spermine N1-acetyltransferase (SSAT), the enzyme responsible for polyamine acetylation (132, 133). Because this process requires acetyl-CoA, polyamine acetylation has been linked to lipid metabolism, which was also elevated in HER2-enriched tumors (134). Additionally, HER2 overexpression is known to activate the PI3K/AKT/mTOR pathway (135), which promotes cell proliferation, survival and lipid synthesis (116, 136). As mTOR activity is closely linked to amino acid availability, including L-arginine, these findings suggest a mechanistic link between oncogenic signaling and altered L-arginine metabolism in HER2 breast cancer. HR+ breast cancers appear to exhibit greater metabolic adaptability, as the estrogen receptor (ER) is linked to multiple key metabolic regulators and signaling pathways, including c-MYC, Ras/Raf/MAPK and PI3K/AKT/mTOR (137). Further studies are needed to decipher the differential regulation of L-arginine metabolism across breast cancer subtypes and explore the links between L-arginine metabolism with other pathways to identify potential therapeutic targets.

## Therapeutic opportunities and clinical trials

Malignant cells rely on L-arginine for anabolic processes including protein and polyamine synthesis, but L-arginine also supports the immune system that targets tumor cells. Several therapeutic strategies have been used to target L-arginine metabolism in breast cancer (**Table 2**). L-Arginine starvation exploits the metabolic vulnerability of cancer cells that depend on extracellular L-arginine due to ASS1-deficiency. Supplementation with L-arginine may support immune cell function and host defense. Moreover, direct targeting of enzymes involved in L-arginine metabolism represents a potential therapeutic strategy. Possible target enzymes include arginases, NOS, CATs, ODC, DDAH, and PRMTs. The following sections describe these strategies in more detail.

**Table 2:** Summary of preclinical and clinical studies targeting L-arginine metabolism in breast cancer.

Type of intervention	Study model	Remarks	References
L-Arginine starvation	*In vitro In vivo*Clinical studies	Induction of mitochondrial distress and autophagy dependent cell death in arginine-auxotrophic cancer cells after treatment with ADI-PEG20Tumor growth suppression in a xenograft model of ASS1-deficient BC following L-arginine deprivation therapy (L-arginine-free diet);L-arginine starvation (L-arginine-free diet) in combination with pharmacological inhibition of JAK suppressed tumor growth in a TNBC mouse modelADI-PEG20 + doxorubicin was safe in ASS1-deficient, HER2-negative BC	(84, 86, 138)(86)(139)(140)
L-Arginine supplementation	*In vitroIn vivo*Clinical studies	Increased tumor cell proliferationEnhanced innate and adaptive immune responses, reduced number of MDSCs, inhibited tumor growth and prolonged survival;Increased tumor growthEnhanced host defense, improved histopathological response rates; increased protein synthesis, Ki67 expression and proliferation	(141)(142)(141)(143–145)
Arginase inhibition	*In vitroIn vivo*	NOHA inhibited cell proliferation and induced apoptosis;TQ increased NO levels, oxidative stress and cell cycle arrest and induced autophagy and apoptosisNor-NOHA suppressed lung metastasis and inhibited carcinogenesis	(39, 95)(146)(147, 148)
NOS inhibition	*In vitroIn vivo*Clinical studies	1400W, L-NMMA and L-NAME reduced BC cell proliferation, migration and mammosphere formation;Flavone reduced cell viability and induced apoptosis;iNOS silencing via c-PTIO inhibited mammosphere formation and enhanced tamoxifen treatment1400W, L-NMMA and L-NAME decreased tumor growth and metastasis,iNOS silencing via c-PTIO enhanced effectiveness of tamoxifen treatmentL-NMMA + taxane lead to 45.8% overall response rate (81.8% LABC; 15.4% metastatic TNBC) and manageable toxicityL-NNMA + docetaxel showed 20% response rate; progression-free survival of 4.5 months; overall survival of 12.8 months	(130)(149)(150)(130, 150–153)(154)(155)
CAT inhibition	*In vitro*	CAT-1 knockdown decreased L-arginine uptake, reduced cell viability and increased apoptosis in ER+ cell lines	(156)
ODC inhibition	*In vitro*Clinical studies	DFMO sensitized TNBC cells to doxorubicin; DFMO with DPCPX as well as ODC-MPI-2 impaired growth and migration of breast cancer cells;NCAO inhibited breast cancer cell growth and migrationPhase 1/2 trial showed limited efficacy of DFMO, but low toxicity	(157–160)(161)
DDAH inhibition	*In vitro*	ZST316 and ZST152 decreased cell migration and vessel-like networks	(162)
PRMT inhibition	*In vitroIn vivo*	MS023 and GSK3368715 (PRMT1 inhibitors) reduced cell viability and increased apoptosis;GSK3368715 showed synergistic effects when combined with chemotherapeutic agents or erlotinib;Genetic knockdown of PRMT1 sensitized TNBC cells to cetuximab treatment;iCARM1 (PRMT4 inhibitor) suppressed breast cancer cell growth;GSK3326595 and JNJ-6461978 (PRMT5 inhibitors) had cytotoxic and antiproliferative effectsGSK3368715 suppressed tumor growth in a TNBC xenograft model;iCARM1 (PRMT4 inhibitor) suppressed breast cancer cell growth;Pemrametostat suppressed tumor growth in combination with fulvestrant	(163, 164)(164)(165)(166)(167, 168)(164)(166)(169)

Clinical studies of PRMT inhibitors are not listed in this table as they have been summarized in detail in a recent review (170). 1400W, N′′[[3-(aminomethyl)phenyl]methyl]-ethanimidamide; ADI-PEG20, pegylated arginine deiminase; ASS, argininosuccinate synthase; BC, breast cancer; CAT, cationic amino acid transporter; c-PTIO, 2-(4-carboxyphenyl)-4,4,5,5-tetramethylimidazoline-1-oxyl-3-oxide; DDAH, dimethylarginine dimethylaminohydrolase; DFMO, difluoromethylornithine; DPCPX, 8-cyclopentyl-1,3-dipropylxanthine; ER+, estrogen receptor-positive; HER2, human epidermal growth factor receptor 2; iCARM1, inhibitor of coactivator-associated arginine methyltransferase 1; iNOS, inducible nitric oxide synthase; JAK, janus kinase; L-NAME, N^5^-[imino(nitroamino)methyl]-L-ornithine methyl ester; L-NMMA, NG-monomethyl-L-arginine; LABC, locally advanced breast cancer; MDSCs, myeloid-derived suppressor cells; NCAO, Nω-chloroacetyl-L-ornithine; NO, nitric oxide; NOHA, Nω-hydroxy-L-arginine; nor-NOHA, Nω-hydroxy-nor-L-arginine; NOS, nitric oxide synthase; ODC, ornithine decarboxylase; ODC-MPI-2, multi-purpose ornithine decarboxylase inhibitor; PRMT, protein L arginine-methyltransferase; TNBC, triple-negative breast cancer; TQ, thymoquinone.

### L-arginine starvation

L-Arginine starvation therapy can be achieved by enzymatic degradation of L-arginine using agents such as pegylated arginine deiminase (ADI-PEG) [reviewed in (171–173)], which metabolizes L-arginine into L-citrulline (but not NO), and a PEGylated recombinant form of human arginase (rhArg-PEG) (174), which converts L-arginine into L-ornithine and urea. rhArg-PEG was reported to inhibit tumor growth and reduce the number and size of metastatic nodules in a breast cancer mouse model (174). Preclinical studies have demonstrated that breast cancer subtypes exhibit variable sensitivity to L-arginine starvation therapy based on their metabolic profiles and ASS1 expression. ASS1-deficient breast cancer cell lines, particularly TNBC cells, showed high sensitivity to L-arginine starvation, which induced autophagy-dependent cell death after 48 h of L-arginine starvation (84). In another study in TNBC, L-arginine starvation also led to reduced cell growth *in vitro*, but not *in vivo* (139). Tishler et al. (139) identified a link between ASS1 and the Janus kinase-signal transducer and activator of transcription (JAK-STAT) pathway, in which ASS1 expression correlates with JAK-STAT gene expression in TNBC. Inhibition of JAK in combination with L-arginine starvation then inhibited the growth of TNBC *in vivo*. Furthermore, Cheng et al. (86) reported that L-arginine starvation for up to 72 h in ASS1-deficient breast cancer cell lines induced aspartate depletion and mitochondrial dysfunction and effectively suppressed tumor growth in a xenograft model. Conversely, HR+ breast cancer cells, which often retain ASS1 expression, demonstrated resistance to L-arginine starvation-induced cytotoxicity (84).

Several clinical studies have investigated L-arginine deprivation therapies, primarily with ADI-PEG20 in ASS1-deficient cancers [reviewed in (89)]. While much of the clinical data come from hepatocellular carcinoma and melanoma, early-phase trials have begun assessing safety and efficacy of ADI-PEG20 in breast cancer patients. A phase 1 study tested ADI-PEG20 in combination with liposomal doxorubicin in ASS1-deficient, HER2-negative breast cancer and confirmed safety and tolerability of the treatment (140). However, resistance to L-arginine starvation therapy remains a major challenge. In ASS1-deficient melanoma cells, for example, L-arginine depletion therapy with ADI-PEG20 led to ASS1 upregulation and consequently to ADI-resistance (175). This was due to activation of the Ras/PI3K/ERK pathway, which then led to c-Myc stabilization and the upregulation of ASS1. When ADI-PEG20 was combined with a PI3K inhibitor in a melanoma mouse model, enhanced antitumor effects were observed compared to the respective monotherapy (175). Combination strategies incorporating ADI-PEG20 with chemotherapy, immune checkpoint inhibitors, or autophagy modulators may offer strategies for overcoming resistance and improving therapeutic efficacy.

### L-arginine supplementation

In a breast cancer mouse model, L-arginine supplementation prolonged survival of the tumor-bearing mice and inhibited tumor growth by enhancing innate and adaptive immune responses and reducing the number of myeloid-derived suppressor cells (142). Brittenden et al. (143) found increased lymphocyte mitogenic reactivity, enhanced natural killer and lymphokine-activated killer cell cytotoxicity after dietary supplementation with 30 g L-arginine per day for three days in breast cancer patients. Conversely, another study has reported increased tumor growth *in vivo* after L-arginine supplementation (141). In this study, mice were fed a diet containing 4% L-arginine for one week prior to implantation of EMT-6 cells and for two weeks thereafter. At two weeks post-implantation, L-arginine-supplemented mice had tumors that were more than twice the size of those in mice fed with basal diet. An early clinical study with 20 breast cancer patients supplemented with 30 g per day L-arginine for three days prior to surgery also indicated that dietary L-arginine supplementation can stimulate breast cancer cell proliferation via increased protein synthesis, which correlated with increased expression of the nuclear activation antigen Ki67 (144). Moreover, a randomized controlled trial including 96 patients with large primary breast cancers found that oral L-arginine supplementation (30 g per day) for three days prior to neoadjuvant chemotherapy improved histopathological response rates in patients with breast tumors <6 cm, indicating a potential enhancement of chemotherapy efficacy (145). However, the potentiating effect of L-arginine supplementation on chemotherapy efficacy was only observed in breast tumors smaller than 6 cm, but not in tumors larger than 6 cm. This may result from better tumor blood flow and drug delivery in small tumors. Large tumors are often poorly vascularized and have hypoxic regions with low proliferation, which makes them less accessible to L-arginine and chemotherapeutic agents (176, 177). These conflicting results highlight the dual role of L-arginine in tumor biology, acting as a nutrient fueling breast cancer cells and as a modulator of immune responses. However, existing studies evaluating L-arginine supplementation in breast cancer patients have not accounted for the molecular heterogeneity of breast cancer subtypes. We hypothesize that these differences may be due to metabolic differences between the intrinsic subtypes of breast cancer. Therefore, further research is essential to unravel potential subtype-specific roles of L-arginine metabolism and to clarify its therapeutic potential in breast cancer treatment.

### Enzyme inhibitors

#### Arginase inhibitors

In preclinical breast cancer research, several studies have investigated arginase inhibitors, which mainly target arginase-2, as it is the predominant isoform expressed in breast cancer cells (39, 94). Inhibition of arginase by N^ω^-hydroxy-L-arginine in a TNBC cell line with high arginase-2 expression induced apoptosis and inhibited cell proliferation (39, 95). This apoptotic effect was reversed by exogenous L-ornithine treatment, indicating that arginase activity supports breast cancer cell growth via the production of L-ornithine and downstream metabolites. In comparison, we observed that siRNA-mediated targeting of arginase-2 resulted in decreased proliferation of TNBC cell lines, whereas proliferation increased in two HR+ cell lines after arginase-2 knockdown. Treatment with L-ornithine reversed these effects. These findings suggest a subtype-specific role of arginase-2 in regulating breast cancer cell proliferation. Another arginase inhibitor, thymoquinone (TQ), was recently reported to inhibit arginase activity in the TNBC cell line MDA-MB-231 (146). In line with our observations, treatment with TQ increased intracellular NO, oxidative stress, cell cycle arrest, and induction of apoptosis and autophagy (146). In an *in vitro* co-culture system of murine macrophages with breast cancer cells, Chang et al. (178) found that macrophage-derived arginase enhanced tumor cell growth by increasing polyamine production and simultaneously suppressed NO-mediated tumor toxicity through decreased NO synthesis. Inhibition of macrophage arginase activity by L-norvaline reversed these effects, demonstrating that arginase inhibition in macrophages can have growth-inhibitory effects on adjacent breast cancer cells.

*In vivo*, arginase inhibition by N^ω^-hydroxy-nor-L-arginine (nor-NOHA) suppressed lung metastasis (147) and inhibited carcinogenesis as evidenced by reduced tumor size and number, lower mortality, improved histopathology, and decreased levels of polyamines and NO (148). Currently, clinical trials directly investigating arginase inhibition in breast cancer patients are very limited. Most existing trials involve arginase inhibitors tested across various solid tumors or include breast cancer only as a part of broader patient cohorts rather than as a primary focus (179–183). One clinical study has evaluated an arginase-1 peptide vaccine in patients with progressive solid tumors, including some breast cancer patients (184). The vaccine was generally well tolerated and induced an immune response, but the study did not provide strong clinical efficacy data for breast cancer. Furthermore, small-molecule arginase inhibitors like OATD-02 and INCB001158, which are potent arginase-1 and arginase-2 inhibitors, respectively, are currently under evaluation in early-phase trials for various cancer types such as colorectal, ovarian, and renal cancers, but breast cancer has not yet been included in these clinical trials (179, 185). INCB001158 was well tolerated with an increase in plasma L-arginine levels in a dose-dependent manner. However, no changes in gene expression or immune activation could be attributed to INCB001158, and the anti-tumor efficacy of INCB001158 was limited, both as a single agent and in combination with pembrolizumab (185). Overall, preclinical data indicate that arginase inhibition has therapeutic potential in breast cancer but the translation into the clinic is still in early stages and has shown limited anti-tumor effects in other tumor entities. Targeting arginase may require patient stratification to identify tumors with high arginase activity and reliance on this pathway.

#### NOS inhibitors

NOS inhibition has emerged as a promising therapeutic strategy in breast cancer with evidence from *in vitro*, *in vivo*, and clinical studies. Preclinical research has extensively investigated the role of iNOS in promoting breast cancer aggressiveness, especially in TNBC, and will be discussed in the following. Selective inhibitors such as N-[[3-(aminomethyl)phenyl]methyl]-ethanimidamide (1400W) and pan-NOS inhibitors like N^G^-monomethyl-L-arginine (L-NMMA) and N5-[imino(nitroamino)methyl]-L-ornithine methyl ester (L-NAME) have been reported to reduce proliferation, migration, and mammosphere formation of TNBC cells *in vitro*, but also showed decreased tumor growth and metastasis in a mouse model of TNBC (130). The mammosphere formation assay is a tool to identify sphere-forming cancer stem cells (186). Cancer stem cells have been associated with cancer progression and recurrence, especially after treatment failure (187). Therefore, mammosphere formation analysis is used to study self-renewal and tumor-initiating capacities. Zhu et al. (149) reported that iNOS inhibition by flavone reduced cell viability and induced apoptosis in breast cancer cells. Studies by López-Sánchez et al. (150) have shown that inhibiting iNOS in ER+ breast cancer cells inhibited mammosphere formation and enhanced the efficacy of tamoxifen treatment *in vitro* and *in vivo*.

Further *in vivo* studies have confirmed these findings. NOS inhibition reduced tumor growth rates, lung metastasis, tumor initiation and enhanced sensitivity to chemotherapeutics (151, 152). Moreover, NO has been implicated in resistance to chemotherapeutics and photodynamic therapy. Fahey et al. (188) demonstrated that iNOS inhibition applied during and after photodynamic therapy improved anti-tumor efficacy in a breast tumor xenograft model. In a more recent study, combined iNOS inhibition via L-NMMA and PI3K inhibition in a metaplastic breast cancer model reduced tumor growth effectively and sensitized chemotherapy-resistant cancer cells to taxane treatment (153). These findings underscore the therapeutic potential of combinatorial strategies.

Although clinical data remain limited, there are early-phase studies available that provide data on NOS inhibition in breast cancer patients. In a phase 1/2 clinical trial with a cohort of 35 patients with metastatic TNBC and chemorefractory, locally advanced breast cancer (LABC), the feasibility of NOS inhibition by L-NMMA in combination with taxane chemotherapy was assessed. An overall response rate of 45.8% was achieved across all patients, with subgroup analyses showing response rates of 81.4% for patients with LABC and 15.4% for patients with metastatic TNBC (155). This study includes a specific cohort of 15 patients with metaplastic TNBC, which was reported separately by Puri et al. (154). In this metaplastic TNBC subgroup, which received a combination therapy of L-NMMA and docetaxel, the overall response rate was 20% and progression-free survival and overall survival were 4.5 months and 12.8 months, respectively (154). Thus, these findings underscore the significance of NOS as a driver of breast cancer progression and treatment resistance. NOS inhibition, alone or in combination with other existing therapies, represents a promising approach to target aggressive breast cancer subtypes.

#### CAT inhibitors

Experimental studies demonstrate that knocking down CAT-1 expression using siRNA leads to decreased L-arginine uptake, reduced cell viability, and increased apoptosis in ER+ breast cancer cell lines (156). These findings indicate that CAT-1 activity is essential for maintaining proliferation in some breast cancer cells, and may be a promising target for disrupting L-arginine dependent metabolic pathways in breast cancer. However, since there are several CAT transporters transporting L-arginine that could potentially compensate for the inhibition of a single transporter, the efficacy of inhibiting a single CAT transporter may be limited. Furthermore, CAT transporters are also responsible for transporting other cationic amino acids, highlighting the potential risk of a broader metabolic disruption. Despite these uncertainties, experimental data are promising and demonstrate the need for further investigation.

#### ODC inhibitors

Several studies have investigated ODC inhibitors in breast cancer. Difluoromethylornithine (DFMO) is an irreversible ODC inhibitor, which has been shown to sensitize TNBC cell lines to cytotoxic chemotherapy with doxorubicin (157). Additionally, DFMO in combination with an A1 adenosine receptor (A1AR) antagonist led to impaired growth and migration of MCF-7 breast cancer cells (158). The treatment with a dual ODC and A1AR inhibitor (ODC-MPI-2) showed similar effects, with a stronger inhibitory effect observed in TNBC cells as compared to T-47D, BT-474 and MDA-MB-453 cells (158). Although a clinical phase 1/2 trial showed limited efficacy of DFMO as a single agent against TNBC, no clinically significant toxicity, and a prolonged growth arrest of liver metastases in one breast cancer patient have been reported (161). Furthermore, N-ω-chloroacetyl-l-ornithine (NCAO) acts as a competitive inhibitor of ODC and was reported to inhibit cell growth and migratory ability of breast cancer cells (159, 160). Overall, ODC inhibitors offer therapeutic opportunities, especially in TNBC, where elevated ODC expression and polyamine synthesis represent metabolic vulnerabilities (157).

#### DDAH inhibitors

Dimethylarginine dimethylaminohydrolase 1 (DDAH1) metabolizes ADMA to L-citrulline and dimethylamine, it has been reported to be upregulated in aggressive TNBC cells (128, 189). DDAH1 can be inhibited by small molecules like ZST316 and ZST152, which act as competitive inhibitors due to their structural similarity to L-arginine (190, 191). *In vitro* studies showed significantly decreased cell migration and tumor vessel-like network formation, without affecting cell viability or proliferation after DDAH1 inhibition by ZST316 and ZST152 in TNBC cells (162). Hence, DDAH1 could serve as a strategy to inhibit tumor progression and metastasis particularly in the highly invasive TNBC subtype. Nevertheless, DDAH inhibition is still in early stages of exploration as a potential therapeutic target in breast cancer, and warrants further research.

#### PRMT inhibitors

Arginine methylation by PRMTs is involved in several cellular processes, such as transcription, RNA metabolism, DNA repair, signal transduction, and protein-protein interactions (64). Over the past decade, therapeutic targeting of PRMTs in breast cancer has been extensively investigated, with a growing body of preclinical data and early clinical investigations. Current research has primarily focused on PRMT1 and PRMT5 inhibitors, as extensively reviewed elsewhere (170, 192).

Preclinical studies demonstrated that PRMT1 inhibition by MS023 and GSK3368715 reduced cell viability and increased apoptosis in breast cancer cells *in vitro* (163, 164). *In vivo*, GSK3368715 significantly suppressed tumor growth in a TNBC xenograft model (164). Moreover, GSK3368715 showed synergistic effects when combined with chemotherapeutic agents or the epidermal growth factor receptor inhibitor erlotinib *in vitro* (164). Consistent with these findings, genetic knockdown of PRMT1 sensitized TNBC cells to cetuximab treatment (165).

Similarly, PRMT5 inhibitors, including GSK3326595 and JNJ-64619178, have demonstrated cytotoxic and antiproliferative effects in breast cancer cell lines (167, 168). The PRMT5 inhibitor pemrametostat synergistically suppresses tumor growth when combined with the selective ER degrader fulvestrant in ER+/RB-deficient cell line- and patient-derived xenografts (169). In addition to PRMT1 and PRMT5, inhibition of PRMT4 by iCARM1 reduced ERα activity in ER+ breast cancer cells both *in vitro* and *in vivo* (166).

Several PRMT inhibitors targeting PRMT5, PRMT4, PRMT7, as well as non-specific agents, are currently progressing through early-phase clinical trials in breast cancer and other solid tumors, as comprehensively reviewed by Wei et al. (170). The present review therefore focuses on preclinical studies relevant to L-arginine metabolism in breast cancer.

## Limitations and future perspectives of therapeutic targets

Taken together, the different therapeutic opportunities discussed above highlight the relevance of targeting L-arginine metabolism in breast cancer. Based on currently available studies, several targets seem to be particularly promising candidates for future clinical development, while others still face limitations. The efficacy of L-arginine starvation therapy using ADI-PEG20 has not yet been proved in breast cancer. Although the application of ADI-PEG20 monotherapy showed limited efficacy in melanoma (193), a phase 2/3 clinical trial in non-epithelioid pleural mesothelioma showed that its combination with chemotherapy resulted in prolonged survival of patients (194). Nevertheless, the development of resistance to ADI-PEG20 remains a major problem, highlighting the need for combinational therapies. In contrast, L-arginine supplementation has not yet demonstrated a clear therapeutic benefit in breast cancer. Since a supplementation of L-arginine can not only enhance antitumor immune responses (142, 143) but may also lead to increased tumor cell proliferation (141), a potential increase in tumor growth remains a major limitation of this therapeutic approach. Therefore, further research is essential to unravel potential subtype-specific roles of L-arginine metabolism and to clarify its therapeutic potential in combination with established treatment strategies in breast cancer.

Preclinical data on arginase inhibition indicate that it has therapeutic potential in breast cancer but the translation into the clinic is still in early stages and has shown limited anti-tumor effects in other tumor entities. Targeting arginase may require patient stratification to identify tumors with high arginase activity and reliance on this pathway. Targeting NOS by using the inhibitor L-NMMA appears to be a particularly promising therapeutic strategy in TNBC. Clinical trials have demonstrated that the combination of L-NMMA with chemotherapy improves survival in TNBC patients (154). In the future combination therapy of NOS inhibitors with PI3k inhibitors or chemotherapy may help to treat chemoresistant TNBC.

Although research on CAT inhibitors in breast cancer remains very limited, this targeting strategy could potentially induce L-arginine starvation and may therefore represent a promising therapeutic target strategy for ASS1-auxotrophic tumors. However, further experimental studies are required to identify suitable inhibitors and to evaluate their therapeutic efficacy and potential off-target effects, before clinical translation. In comparison, the development of ODC inhibitors is already more advanced, with data from preclinical and clinical studies. Although treatment with DFMO alone showed limited efficacy in TNBC in an early clinical trial (161), data of a preclinical study suggest a synergistic effect of ODC inhibitors when combined with other therapeutic approaches (157, 158). Further studies are needed in the future to better understand subtype-specific differences in treatment success and to evaluate the efficacy of combination therapies *in vivo*.

*In vitro* studies have shown that DDAH inhibitors can have an inhibitory effect on cell migration in TNBC (162), highlighting the potential of this therapeutic approach. Nevertheless, further studies are needed to investigate its effectiveness *in vivo*. As Wei et al. (170) have already discussed in detail, the inhibition of PRMT5 in particular shows great promise and is currently being evaluated in clinical trials. However, the development of additional specific PRMT inhibitors is of great importance to minimize adverse effects. Overall, targeting the L-arginine metabolism offers promising therapeutic strategies in breast cancer and may provide new opportunities for personalized treatment options. However, the current literature suggests that combining these strategies with established treatment approaches may be more effective than targeting L-arginine metabolism alone.

## Discussion and conclusion

L-Arginine plays a critical role in breast cancer biology by serving as a substrate for key metabolic pathways, involving enzymes such as nitric oxide synthase and arginases. L-Arginine metabolism influences tumor growth, immune regulation, and cellular signaling, making it an attractive target for cancer therapy. Breast cancer is characterized by heterogeneity with distinct metabolic phenotypes across the different subtypes. While there is emerging data that suggest differential regulation of L-arginine metabolism across breast cancer subtypes, existing studies remain limited, and sometimes contradictory. Preclinical studies underscore the essential role of L-arginine metabolism in breast cancer progression and immune modulation, but clinical evidence is still limited. Although strategies like L-arginine deprivation and modulation of metabolic pathways appear promising in preclinical studies, translation into effective clinical therapies requires further validation. Further studies are needed to better understand the subtype-specific role of the L-arginine metabolism, which may support the development of more individualized treatment options.
